# Early outcomes of the arthroscopic Latarjet procedure in a series of 37 patients with shoulder instability

**DOI:** 10.1186/s12891-021-04726-3

**Published:** 2021-10-02

**Authors:** Zheng Zeng, Chuan Liu, Yang Liu, Yan Huang

**Affiliations:** 1 Department of Orthopaedic Surgery, Beijing Tiantan Hospital, Capital Medical University, Beijing, 100070 China; 2 Department of Orthopaedic Sports Medicine, the 7th center of PLAGH, Beijing, 100700 China

**Keywords:** Recurrent anterior shoulder dislocation, Arthroscopic Latarjet, Active patients, Glenoid bone loss

## Abstract

**Background:**

Anterior shoulder dislocation remains a clinical challenge. This study aimed to assess the graft position and clinical outcomes of the arthroscopic Latarjet procedure and capsular repair for the treatment of recurrent anterior shoulder dislocation with significant glenoid bone loss in 37 patients.

**Methods:**

Between 2017 and 2017, 37 patients underwent arthroscopic Latarjet plus capsular repair procedure for recurrent anterior shoulder dislocation combined with significant glenoid bone loss. In follow-up examinations, Walch-Duplay scores, subjective shoulder value (SSV) scores, Rowe scores, and active range of motion (AROM) were assessed. Three-dimensional computed tomography (CT) was used to evaluate coracoid graft position and bone resorption. A new method of evaluating the position of the coracoid bone block after Latarjet (H-Z method) was developed.

**Results:**

Thirty-seven patients were included in this study. Follow-up ranged from 6 to 36 months postoperatively (with an average of 13 months). No recurrent dislocation occurred at the final follow-up, and there was no significant effect on the AROM (all *p* > 0.05). Rowe (from 42.2 ± 5.6 to 91.1 ± 3.3), Walch-Duplay (from 31.5 ± 8.0 to 92.6 ± 3.7), and SSV (from 63.9 ± 6.1 to 79.3% ± 5.0%) scores were improved significantly after surgery (all *p* < 0.001). CT showed that the 29 patients had varying degrees of bone resorption, and 23 recovered to the preinjury level of motional function within 6–12 months after surgery.

**Conclusions:**

In active patients with recurrent anterior shoulder dislocations and significant glenoid bone loss, the arthroscopic Latarjet procedure plus capsular repair could restore shoulder stability satisfactory.

## Background

The incidence of anterior shoulder dislocation in the general population is approximately 23.9/100,000 per year, and its recurrence rate is also high, especially in patients younger than 20 years [1]. Repeated shoulder dislocation could result in bony deficiency at the glenoid and/or humeral head due to recurrent avulsion, compression, and wear of the bone [2].

Arthroscopic glenolabral capsular repair (Bankart surgery) is the most common surgical method for treating recurrent anterior shoulder dislocation. In particular, anterior shoulder dislocation accompanied by significant glenoid bone loss has a recurrence rate of 67% after Bankart surgery and 89% in athletes participating in contact sports [3]. Therefore, patients with glenoid bone loss above 15% and other significant risk factors are treated with the Latarjet procedure [4], which was firstly reported by Dr. Latarjet in 1954 [5].

In the past 20 years, an increasing number of patients have undergone open Latarjet surgery for anterior shoulder instability [6]. The open Latarjet approach for anterior shoulder instability has good clinical efficacy, as evidenced by 86% of patients showing excellent or good functional scores and 90% who were satisfied with surgical outcomes during an average follow-up of 16 years [7]. Still, open surgery has various disadvantages, including large incisions, difficulty in exposing the operation field, and multiple surgical complications. In 2007, Lafosse et al. [8] firstly reported an all-arthroscopic Latarjet surgery, characterized by smaller surgical trauma, fewer complications, and faster postoperative recovery. Since then, this technique has been increasingly used [8, 9]. In addition to coracoid bone grafting in the Latarjet procedure, Zhu et al. [10] suggested the repair and reconstruction of the anterior articular capsule for increasing anterior shoulder stability and preventing direct collision between the humeral head and the coracoid process. Reports assessing the all-arthroscopic operation are limited due to the relatively high difficulty and short time of clinical application of this surgical technique [8–14].

Therefore, this study aimed to evaluate the graft position and clinical outcomes of the arthroscopic Latarjet procedure and capsular repair. The position of the coracoid bone block was measured using a new method.

## Methods

This retrospective study assessed patients treated with the all-arthroscopic Latarjet procedure plus capsular repair for recurrent anterior shoulder dislocation between 2017 and 2019. Inclusion criteria were age ≤ 40 years, preoperative CT scan, showing significant glenoid bone loss greater than 15% compared to the healthy side (both sides were investigated using 3D reconstruction). The exclusion criteria were revision surgery for shoulder instability, multi-directional instability of the shoulder, and concomitant injuries such as ipsilateral rotator cuff injury. Demographic information, affected shoulder, frequency of dislocations, surgery information, and preoperative functional evaluation, including Walch-Duplay scores, subjective shoulder value (SSV) scores, Rowe scores, and the active shoulder range of motion (AROM) [15–18], were exacted.

This study was approved by the Ethical Research Committee of Beijing Tiantan Hospital and the 7th center of The General Hospital of the People’s Liberation Army. Informed consents were waived due to the retrospective nature.

### Surgical procedure

The arthroscopic Latarjet procedure was performed as previously described [9–11]. The patient was placed in the beach-chair position and underwent general anesthesia. Systolic blood pressure was strictly controlled at approximately 90 mmHg, and the muscles were completely relaxed perioperatively. For the surgical procedure, the arthroscope and a probe hook were inserted through portals A and E, respectively, for a comprehensive assessment of bone loss, soft tissue damage, dynamic stability, and other conditions of the shoulder joint. Subsequently, a shaver was inserted through portal D to fully expose the posterior coracoid process. After reshaping the coracoid process tip and decorticating the posterior surface of the coracoid, a radiofrequency scalpel was placed through portal E to remove the residual glenoid labrum and avulsed bone, separating the adhered joint capsule downward to 6 o’clock. After the joint capsule was detached, the glenoid bone bed was smoothened.

In the second stage, the arthroscope was inserted through portal D to fully expose the coracoid process and the conjoined tendon. After the medial soft tissue of the conjoined tendon was detached to expose the musculocutaneous nerve, the coracoacromial ligament and the pectoralis minor muscle were severed at the insertion of the coracoid process. The brachial plexus nerves, especially the axillary nerve, were visualized.

In the third stage, a drill was made perpendicular to the upper surface of the coracoid through portal H to create a tunnel. A guidewire was inserted into the bone tunnel, with its end exiting from a double-lumen cannula placed through portal M. After bone grooves were generated on the anterior and inferior surfaces of the coracoid through portal E, a coracoid osteotomy was performed through portal H. The coracoid was then fixed on the double-lumen cannula, followed by hemostasis of the base of the coracoid process with bone wax.

The fourth stage included coracoid transfer and bone graft fixation. The subscapularis muscle and part of the tendon were split at the level of the 4:30 position of the glenoid. While lifting the middle and upper parts of the subscapularis muscle with an Ethibond wire, the prepared coracoid bone block was placed on the glenoid bone bed of the shoulder through the split subscapularis muscle. The direction of the screw was parallel to the glenoid as much as possible. After guide pin insertion and the creation of two drill holes, two 4.5-mm cannulated screws were used to fix the coracoid bone block. Through portal A, a bur was used to ensure that the lateral rim of the coracoid bone block was at the same level or slightly below the glenoid surface. Finally, the arthroscope was placed through portal A, and the anterior capsule was sutured. Posterosuperior humeral head bone loss was left untreated.

### Postoperative rehabilitation

Fist making/release exercises and wrist activities were encouraged immediately after operation. Passive elbow flexion and extension were added the day after surgery. Passive shoulder movements were allowed 3 to 5 days post-surgery. Codman’s pendulum exercises started 2 weeks after surgery. After 4 weeks, shoulder motion exercises were gradually added. Active elbow flexion was avoided within the first 6 weeks. After 6 to 8 weeks, daily activities below the shoulder level were resumed, and progressive resistance training (except the use of biceps) was started. After a 3-month postoperative CT examination, normal daily and sports activities were allowed. High-intensity exercises requiring bending of the elbow and upper limbs against a resistive force were to be avoided for 6 months.

### Evaluation indices at postoperative follow-up

The patients were routinely followed up at 1, 2, 3, 6, and 12 months after surgery and once per year afterward. During follow-up, the patients underwent X-ray and CT scan with 3D reconstruction. Walch-Duplay scores, SSV scores, Rowe scores, and AROM were recorded to evaluate postoperative shoulder function.

**Fig. 1:**
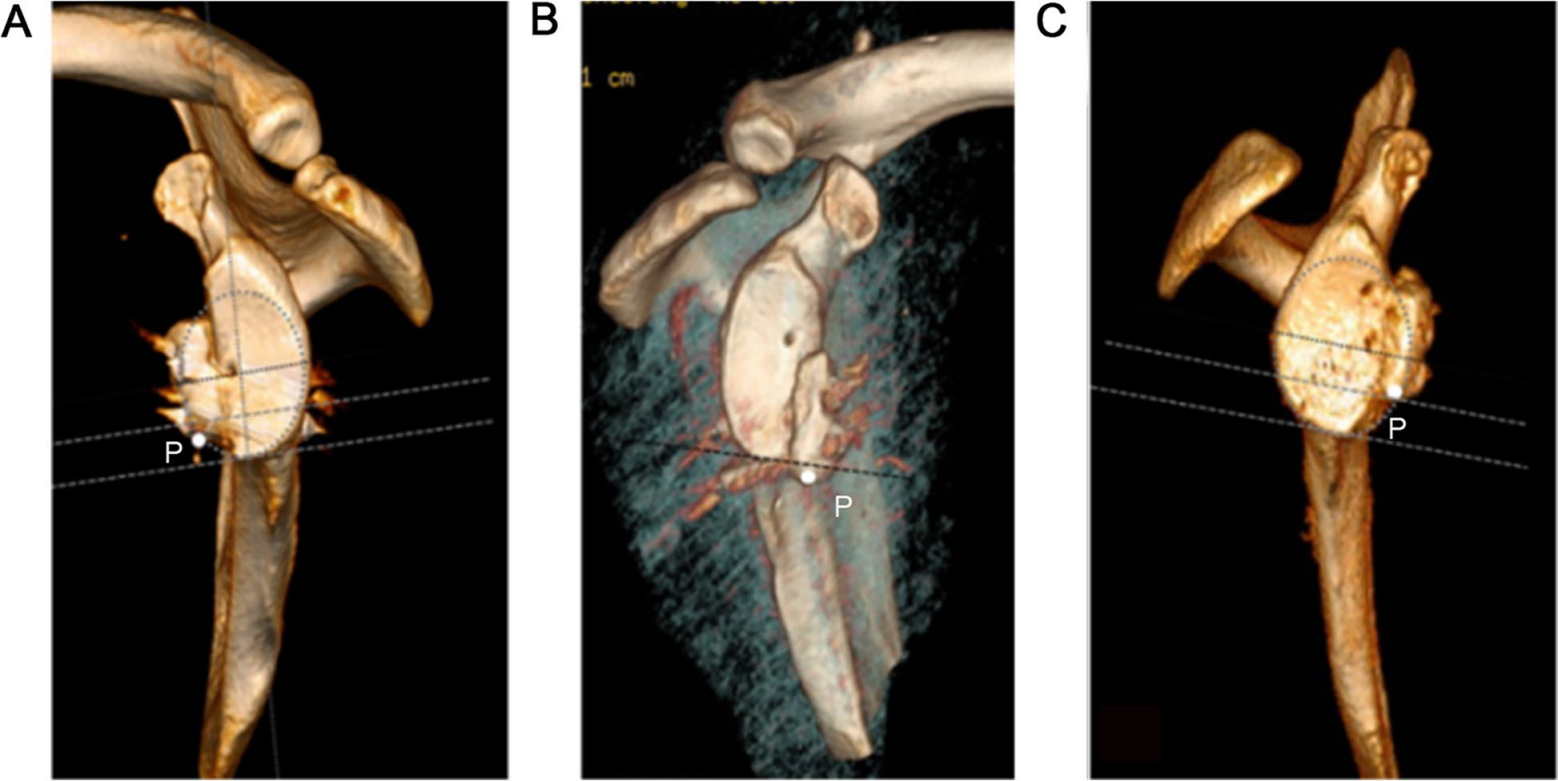
H-Z method for bone block height assessment. **A** The apex of the coracoid process (point P) is located between the 4 o’clock and 6 o’clock lines on the oval, indicating that the bone graft is in the correct position. **B** Point P is located below the 6 o’clock line, indicating too low bone graft position. **C** Point P is located above the 4 o’clock line, indicating too high bone graft position

On a two-dimensional cross-section, lateral deviation of the coracoid bone graft was identified with the lateral edge of the graft exceeding the articular surface of the glenoid by more than 3 mm; medial deviation was considered with the lateral edge of the graft being medial to the rim of the glenoid articular surface by more than 5 mm [12]. Correct bone block height was observed from the en face view of the 3D CT reconstructed glenoid image. We developed a new evaluation method, termed the H-Z method. In this technique, the lowest point P of the coracoid bone block is considered the reference point. We drew an oval in the front-view image of the glenoid, the longitudinal axis (the connection between the highest and lowest points of the glenoid) through the center of the oval, and the vertical line of the longitudinal axis along with the 4 o’clock and 6 o’clock planes. Point P above the 4 o’clock plane was defined as too high, and point P below the 6 o’clock plane as too low (Fig. 1).

The bone resorption status of the coracoid bone graft was evaluated at 6 months and classified into four grades for assessment based on postoperative CT scan [19]: Grade 0 (no resorption; cone of the screw head buried in the coracoid bone graft); Grade I (minor resorption; only the screw head exposed outside the bone graft, with the whole screw shaft inside the bone); Grade II (major resorption; part of the screw shaft exposed outside the graft, with some bone still left on the glenoid neck); Grade III (total resorption; screw head and shaft both totally exposed, with all of the coracoid bone graft absorbed and no bone left on the glenoid neck).

### Statistical analysis

The SPSS 24.0 statistical software (IBM Corp., Armonk, NY) was used for data analysis. The normality of the distribution of the continuous variables was tested using the one-sample Kolmogorov-Smirnov test. Continuous variables with a normal distribution were presented as mean (standard deviation [SD]); non-normally distributed variables were reported as median (range). Paired *t*-test was performed for pre- and post-operation comparisons. *P* < 0.05 indicated a statistically significant difference.

## Results

**Fig. 2:**
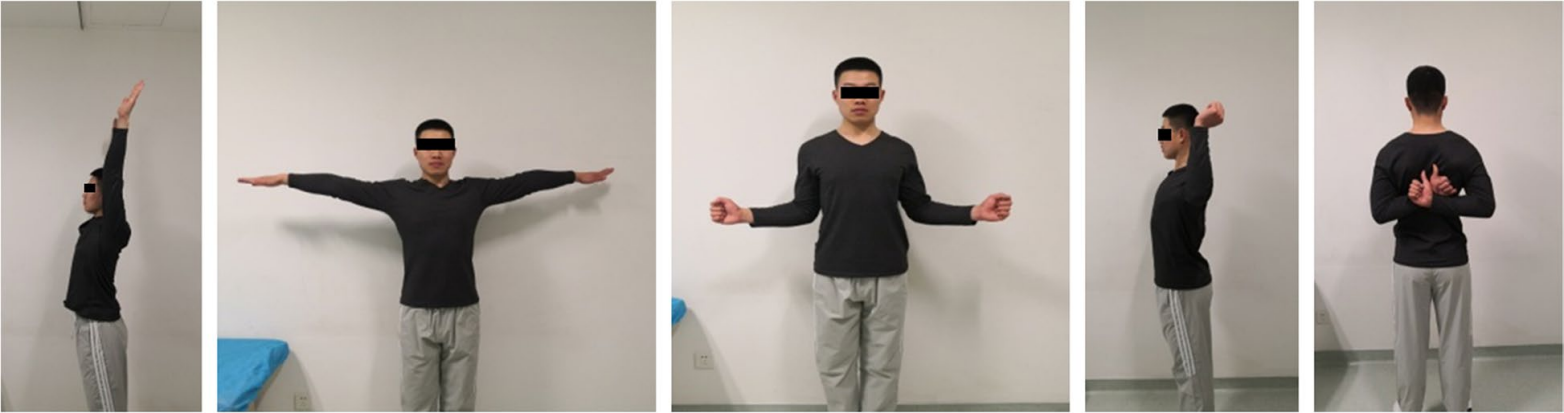
Left shoulder functional status was remarkably improved 10 months after the Latarjet procedure

This study included 37 patients (Table 1). All patients were individuals with an active daily life. The patients were followed for a median of 13 (IQR: 8–22) months, with a range of 6 to 36 months (Table 1). At the final follow-up, none of the 37 patients had recurrent dislocation after surgery. Meanwhile, 23 (62.2%) patients recovered to the preinjury shoulder function level (Fig. 2), and 14 (37.8%) had an improved functional level 6 to 12 months after surgery.

**Table 1:** General information

Characteristics	Subjects (*N* = 37)
Sex (male/female), n/n	37/0
Age (years), mean ± SD	25.4 ± 4.9
Affected shoulder (left/right), n/n	18/19
Frequency of dislocations (times), median (IQR)	24 (10–56)
Duration from first shoulder dislocation to the time of surgery (months), median (IQR)	41 (19–82)
First surgery/revision surgery, n/n	29/8
Operating time (minutes), mean ± SD	123.7 ± 60.3
Follow-up period (months), median (IQR)	13 (8–22)

*SD* Standard deviation, *IQR* Interquartile range

The day after the operation, a CT scan did not reveal bone block fragmentation and screw head or body exposure in any patients. The grafted bone position (in mm) was too low in four patients (13.5%) and too high in one (2.7%). The coracoid bone graft rim was in line with the glenoid rim in 35 patients (94.6%) and laterally deviated in two (5.4%); no patient had a medially deviated bone graft.

Bone resorption was evaluated at 6 months. Of the 37 patients in the present study, 29 (78.4%) had bone resorption to varying degrees, including 15 cases at the proximal site, 13 at both ends, and one at the distal site. The bone surrounding the upper screw showed Grade I resorption in 22 patients (59.5%) and Grade II resorption in six (16.2%); the bone surrounding the lower screw showed Grade I resorption in 12 patients (32.4%) and Grade II resorption in two (5.41%); no patient had Grade III bone resorption.

Comparing preoperative and final follow-up data, no statistically significant differences in shoulder anteflexion, lateral rotation with the elbow against the body, medial rotation, and lateral rotation at 90 degrees of abduction were detected, but statistically significant differences were noted in Rowe (42.2 ± 5.6 versus 91.1 ± 3.3; *P* < 0.001), Walch-Duplay (31.5 ± 8.0 versus 92.6 ± 3.7; *p* < 0.001), and SSV (63.9% ± 6.1% versus 79.3% ± 5.0%; *p* < 0.001) scores (Table 2).

**Table 2:** AROM and functional scores before and after surgery

Variables	Mean ± SD	Final follow-up	*t* value	*P* value
	Preoperative			
**Shoulder AROM**				
Anteflexion	166.4° ± 7.1°	164.2° ± 7.4°	*t* = 1.554	0.129
Lateral rotation with the elbow against the body	54.7° ± 6.1°	53.5° ± 6.3°	*t* = 1.271	0.212
Medial rotation (vertebral level)	9.4 ± 1.4	9.2 ± 1.0	*t* = 0.770	0.446
Lateral rotation at 90 degrees of abduction	71.6° ± 6.1°	73.1° ± 5.9°	*t* = −1.264	0.214
**Functional score**				
Rowe score	42.2 ± 5.6	91.1 ± 3.3	*t* = −43.319	0.001
Walch-Duplay score	31.6 ± 8.0	92.6 ± 3.7	*t* = −41.516	0.001
SSV (%)	63.9 ± 6.1	79.3 ± 5.0	*t* = − 13.561	0.001

*AROM* Active range of motion, *SSV* Subjective shoulder value

### Complications

During follow-up, one patient (2.7%) presented transient musculocutaneous nerve injury and local hematoma. The hematoma was absorbed 2 weeks later, and the observed musculocutaneous nerve injury was completely relieved 3 months after surgery. Two patients (5.4%) had cortical bone dehiscence along the lower screw canal. One patient (2.7%) showed early osteoarthritis on CT images but no obvious symptoms at the 1-year follow-up visit.

## Discussion

This study showed that the arthroscopic Latarjet procedure plus an anterior capsular repair could achieve satisfactory short-term outcomes in active patients with an anterior shoulder dislocation and significant bone loss. Lateral rotation at 90° of abduction of the injured shoulder was improved after the operation. All the grafted coracoid bone blocks healed, but many patients had bone resorption. In this study, all patients were males of 18–36 years of age. All patients, including 28 soldiers, were young people with an active daily life. All patients had multiple dislocations and shoulder instability, but the postoperative evaluation was good.

Currently, the Latarjet procedure is the most common treatment option for shoulder joint instability accompanied by significant bone defects and has shown satisfactory outcomes [20, 21]. In this study, all patients were under 40 years old, and most were extremely active soldiers in daily life. After surgical treatment, shoulder function was recovered to the preinjury level in 23 of the 37 patients and improved in the remaining 14. This recovery rate was lower than previously reported. Stirma et al. reported a successful Latarjet procedure in all professional athletes, with no complications [22]. Maman et al. reported that treatment with the Latarjet procedure results in redislocation and subluxation rates of 3.7 and 14.8%, respectively, with a mean SSV score of 81.5 (range, 40–100) [21].

At present, there are controversies regarding the method for assessing the position of the grafted coracoid bone block after the Latarjet procedure. Whether the bone block is laterally or medially deviated is mostly determined by a method proposed by Kany et al. [12]; however, methods for evaluating bone block height vary widely. Lafosse et al. [14] suggested that the ideal bone block position is at 3–5 o’clock. Boileau et al. [11] suggested that the coracoid bone block should be positioned lower than the horizontal midline of the glenoid. Zhu et al. [10] stated that the bone graft is too high with the midpoint of its long axis above the horizontal midline and too low with the midpoint below the lower rim of the glenoid. The first two methods presented above provide relatively rough estimates, while the third provides quantitative standards for a more accurate assessment. In this study, regardless of coracoid bone graft size, the measurement point was the tip of the coracoid process, which is at the same position as the midpoint of the conjoined tendon. According to a study by Nourissat et al. [23], the bone block at 4 o’clock of the glenoid has the best “blocking” outcome. Of the seven malpositioning cases (18.9%) in this study, the grafted bone was too low in four patients and too high in one; in the remaining two cases, the coracoid bone graft rim was laterally deviated. It should be noted that these malposition cases were mainly in the early period as the technique was being refined. The above rate is still low compared with that of the open technique for which malpositioning rates of 20–40% have been reported [24].

Bone resorption remains a major concern after the Latarjet procedure. Of the 37 patients in the present study, 29 (78.4%) had bone resorption to varying degrees, including 15 cases at the proximal site, 13 at both ends, and one at the distal site. According to Zhu et al. [10], the incidence of bone resorption was 82.7%, including 28.8% of subjects with significant resorption (grades II and III), 1 year after the arthroscopic Latarjet procedure. Extremely high rates of bone resorption have been reported after the Latarjet procedure, i.e., 90.5 and 100% by Zhu et al. [19] and Di Giacomo et al. [25], respectively, indicating relatively lower values in the present study. The bone surrounding the proximal screw is significantly more likely to be resorbed than that surrounding the distal screw, which may be related to blood supply [19] or postoperative bone remodeling [13].

Osteolysis is influenced by biological and biomechanical factors. Moroder et al. [26] observed anatomic graft remodeling in patients with significant preoperative glenoid bone loss who were treated with iliac crest bone grafting. Mechanotransduction, therefore, helps to maintain the graft in those areas that are subject to contact pressure and shear forces. The lack of mechanical stimuli in certain areas of the graft, in contrast, may contribute to osteopenia and bone resorption in these areas [27]. Thomas et al. [28] reported that there is no difference between the Bristow procedure and the conjoined tendon transfer alone in restoring anterior translation. Di Giacomo et al. [25] reported that there was a significant amount of osteolysis of the coracoid bone graft in all patients regardless of the size of the glenoid bone defect addressed. In addition to eliminating bone resorption caused by infection, ischemia, and poor healing, bone resorption caused by biomechanical factors is actually a process of bone remodeling. In this study, bone resorption was observed in all patients at the final follow-up, but there was no instability. This supports the idea that the bone block itself does not seem to be the principal factor for stabilization. The stabilizing effect of the Latarjet procedure must be due to other components of the technique, such as the sling effect and capsular effect. It will have to be specifically examined in future studies.

This study had some limitations. First, it is a small sample. Secondly, the follow-up was relatively short, necessitating further analysis of long-term follow-up data regarding shoulder function recovery and bone resorption.

## Conclusions

In active patients with recurrent anterior shoulder dislocation combined with significant bony deficiencies, the arthroscopic Latarjet procedure plus capsular repair shows satisfactory short-term outcomes. The new H-Z method for evaluating the position of the coracoid bone block after Latarjet could be a valuable tool to evaluate the outcomes of Latarjet. The causes of bone resorption and long-term functional outcomes require further research and evaluation.

## Data Availability

All data generated or analyzed during this study are included in this published article.

## Abbreviations

SSV: Subjective shoulder value
AROM: Active range of motion
CT: Computed tomography
SD: Standard deviation
IQR: Interquartile range
